# Long-term Follow-up of 84 Patients With Giant Prolactinomas—A Swedish Nationwide Study

**DOI:** 10.1210/clinem/dgad393

**Published:** 2023-07-04

**Authors:** Christos Himonakos, Pia Burman, Henrik Borg, Per Dahlqvist, Britt Edén Engström, Bertil Ekman, Louise Emilsson, Daniel S Olsson, Oskar Ragnarsson, Jeanette Wahlberg, Anna-Karin Åkerman, Charlotte Höybye, Katarina Berinder

**Affiliations:** Department of Molecular Medicine and Surgery, Karolinska Institute, 171 76, Stockholm, Sweden; Department of Internal Medicine, Center for Endocrinology and Diabetes, Karlstad Central Hospital, 651 85, Karlstad, Sweden; Department of Endocrinology, Skåne University Hospital, Lund University, 214 28, Malmö, Sweden; Department of Endocrinology, Skåne University Hospital, Lund University, 222 42, Lund, Sweden; Department of Public Health and Clinical Medicine, Umeå University, 901 87, Umeå, Sweden; Department of Medical Sciences, Endocrinology and Mineral Metabolism, Uppsala University and Uppsala University Hospital, 751 85, Uppsala, Sweden; Department of Endocrinology and Department of Health, Medicine and Caring Sciences, Linköping University, 581 83, Linköping, Sweden; Department of General Practice, Institute of Health and Society, University of Oslo, 0318, Oslo, Norway; Nysäter Health Care Center and Center for Clinical Research, County Council of Värmland, 651 85, Karlstad, Sweden; Department of Medical Epidemiology and Biostatistics, Karolinska Institute, 171 77, Stockholm, Sweden; Department of Endocrinology at Sahlgrenska University Hospital, 413 45, Gothenburg, Sweden; Department of Internal Medicine and Clinical Nutrition, Institute of Medicine at Sahlgrenska Academy, University of Gothenburg, 413 45, Gothenburg, Sweden; Cardiovascular, Renal and Metabolism, BioPharmaceuticals R&D, AstraZeneca, 430 51, Gothenburg, Sweden; Department of Endocrinology at Sahlgrenska University Hospital, 413 45, Gothenburg, Sweden; Department of Internal Medicine and Clinical Nutrition, Institute of Medicine at Sahlgrenska Academy, University of Gothenburg, 413 45, Gothenburg, Sweden; Department of Medicine, Örebro University Hospital, 701 85, Örebro, Sweden; School of Medical Sciences, Faculty of Medicine and Health, Örebro University, 701 82, Örebro, Sweden; Department of Molecular Medicine and Surgery, Karolinska Institute, 171 76, Stockholm, Sweden; Department of Medicine, Örebro University Hospital, 701 85, Örebro, Sweden; Department of Molecular Medicine and Surgery, Karolinska Institute, 171 76, Stockholm, Sweden; Department of Endocrinology, Karolinska University Hospital, 171 76, Stockholm, Sweden; Department of Molecular Medicine and Surgery, Karolinska Institute, 171 76, Stockholm, Sweden; Department of Endocrinology, Karolinska University Hospital, 171 76, Stockholm, Sweden

**Keywords:** giant prolactinomas, dopamine agonists, dopamine agonist resistance, long-term follow-up, Ki-67

## Abstract

**Purpose:**

To describe the clinical presentation and treatment outcomes in a nationwide cohort of patients with giant prolactinomas.

**Methods:**

Register-based study of patients with giant prolactinomas [serum prolactin (PRL) > 1000 µg/L, tumor diameter ≥40 mm] identified in the Swedish Pituitary Register 1991-2018.

**Results:**

Eighty-four patients [mean age 47 (SD ±16) years, 89% men] were included in the study. At diagnosis, the median PRL was 6305 µg/L (range 1450-253 000), the median tumor diameter was 47 mm (range 40-85), 84% of the patients had hypogonadotropic hypogonadism, and 71% visual field defects. All patients were treated with a dopamine agonist (DA) at some point. Twenty-three (27%) received 1 or more additional therapies, including surgery (n = 19), radiotherapy (n = 6), other medical treatments (n = 4), and chemotherapy (n = 2). Ki-67 was ≥10% in 4/14 tumors. At the last follow-up [median 9 years (interquartile range (IQR) 4-15)], the median PRL was 12 µg/L (IQR 4-126), and the median tumor diameter was 22 mm (IQR 3-40). Normalized PRL was achieved in 55%, significant tumor reduction in 69%, and combined response (normalized PRL and significant tumor reduction) in 43%. In the primary DA-treated patients (n = 79), the reduction in PRL or tumor size after the first year predicted the combined response at the last follow-up (*P* < .001 and *P* = .012, respectively).

**Conclusion:**

DAs effectively reduced PRL and tumor size, but approximately 1 patient out of 4 needed multimodal treatment. Our results suggest that the response to DA after 1 year is useful for identifying patients who need more careful monitoring and, in some cases, additional treatment.

Prolactinomas are the most common pituitary tumors, with an annual incidence of 1.6 to 2.2 per 100 000 inhabitants (1-3). The tumors are classified, according to their maximum diameter, as microprolactinomas (<10 mm) or macroprolactinomas (≥10 mm). A subset of patients with macroprolactinomas have tumors ≥40 mm, termed giant prolactinomas (4, 5). Giant prolactinomas account for approximately 2% to 4% of all prolactinomas (4, 6), are characterized by significant extrasellar extension, and are usually defined as a maximum tumor diameter of ≥40 mm in association with prolactin (PRL) >1000 µg/L (4, 5).

Macroprolactinomas, including giant prolactinomas, occur predominantly in men (7), in contrast to microprolactinomas, which are more common in women (1, 8). Macro- and giant prolactinomas in men are more often invasive, have higher Ki-67 and other markers of proliferation (7, 9, 10), and more commonly demonstrate aggressive growth (11). Giant prolactinomas often cause typical symptoms due to hyperprolactinemia per se such as hypogonadism and galactorrhea (12-14). However, because of their large size, the most striking symptoms at diagnosis are often caused by the mass effect of the tumor, such as headache, visual field defects (VFD), other neurological symptoms, and pituitary insufficiencies (12-14).

Dopamine agonists (DA) are the first-line treatment for prolactinomas and lead to inhibition of PRL production and secretion, improved gonadal function, and reduced tumor size (15). In a review of 12 studies of 309 patients with macroprolactinomas, including giant prolactinomas, treatment with cabergoline (CAB) was accompanied by normalization of PRL in 80% and significant tumor reduction in 87% of the patients, thus demonstrating a good response to DA even in larger tumors (16). In many patients with giant prolactinomas, DAs rapidly reduce tumor size (4), although with increasing tumor size the proportion of patients achieving normalized PRL decreases (17). Giant prolactinomas may therefore pose a therapeutic challenge and require additional treatment modalities, including surgery, radiotherapy, and/or chemotherapy (13, 14, 18-21).

Due to the rarity of giant prolactinomas, limited information is available on their clinical course, and the 2 largest studies thus far have included 47 and 71 patients (13, 14). The aim of the present nationwide study was to describe baseline characteristics, treatment strategies, and outcomes during long-term follow-up in a large cohort of patients.

## Materials and Methods

### The Swedish Pituitary Register

The Swedish Pituitary Register (SPR) was established in 1991. It is a nationwide register run by the Regional Cancer Center (Stockholm-Gotland) and is financially supported by the Swedish government. The SPR collects prospectively registered information of patients with pituitary adenomas, craniopharyngiomas, and cysts. The SPR is organized by a multidisciplinary team of specialists from all 6 health care regions in Sweden.

### Ethics Approval

This study was performed in line with the principles of the Declaration of Helsinki. SPR was approved by the Ethics Committee, Karolinska Institute, Stockholm, Sweden (Dnr 2003/515/03 and Dnr 2012/915-32). Since the SPR is a quality register supported by the government, a written approval for inclusion in the register is not requested but the patients must be informed, with an opt-out possibility, either orally or by written information. Some data were not complete in the SPR, and a separate ethical approval granted by the Swedish Ethical Review Board (Dnr 2019-03461) for the present study allowed us to include missing data from medical records without additional consent.

### Study Design and Case Findings

Patients diagnosed with a prolactinoma between December 1991 and December 31, 2018, were identified in the SPR (n = 1897). The criteria for classification as a giant prolactinoma were serum PRL concentrations >1000 µg/L (21 200 mU/L), a tumor size of 40 mm or more in at least 1 dimension, and absence of concomitant other hormonal secretion (4). The medical records were reviewed by experienced endocrinologists. A specific form was used to collect information on clinical presentation, PRL concentrations, pituitary function, tumor size, ophthalmologic examination, treatment modalities, maximum doses of DAs, histopathological data (Ki-67, PRL, and other hormonal immunoreactivity), follow-up time, and outcomes of treatment. The treating physician decided on the DA drug to use, the start dose, and dose adjustments when considered appropriate. The time interval for follow-up at 6 months, 1 year, and 2 years was ±3 months; for follow-up at 5 years and 10 years it was ±1 year.

### Hormonal Assays and Imaging

Serum PRL and other hormones were analyzed locally with commercially available methods that are in routine use and their corresponding reference ranges were applied. The serum PRL concentrations, expressed as mU/L, were converted to µg/L by dividing by a conversion factor of 21.2 (22). Pituitary deficiencies were diagnosed according to the international guidelines (23). Stimulation tests for GH deficiency were not consistently performed, and thus, the total number of patients with GH deficiency is unknown. Magnetic resonance imaging (MRI) or computed tomography (CT) was performed at diagnosis and during follow-up.

### Follow-up and Response to Therapy

The last follow-up was defined as the last documented PRL or MRI of the pituitary (whichever was the latest). The last documented follow-up was in March 2020. Final PRL was the last documented serum PRL at follow-up. Normalization of PRL was defined as a serum PRL concentration within the normal reference range for the specific assay used at the last follow-up. The proportion of patients with PRL concentrations <2 × upper level of normal (ULN) at the last follow-up was calculated. Tumor response was defined as a ≥30% decrease in the maximum tumor diameter at the last performed MRI, according to the RECIST definition (24). Tumor enlargement was defined as a ≥20% increase in the sum of the accessible diameters (24). A combined response was defined as both normalization of serum PRL and tumor response as described earlier.

### Statistical Methods

Statistical analysis was performed using IBM SPSS Statistics, version 26. Data are expressed as the median and range (min-max) or interquartile range (IQR) for nonnormally distributed data and otherwise as the mean (±SD). Differences in normally distributed quantitative variables were calculated with Student's t test; otherwise they were calculated by the Mann-Whitney U test. Categorical data are presented as absolute numbers or percentages; differences between the groups were evaluated with the chi-square or Fisher's exact test. The correlation between serum PRL concentrations and maximum tumor diameter was estimated using Spearman´s correlation. Within-group differences were evaluated with the Wilcoxon signed-rank test. Multivariate analysis of serum PRL at diagnosis, maximum tumor diameter at diagnosis, multimodal treatment, reduction of serum PRL, and maximum tumor diameter 1 year after diagnosis associated with combined response at last follow-up as the outcome variable was performed with binary logistic regression. A *P*-value <.05 was considered significant.

## Results

### Characteristics at Diagnosis

Eighty-four patients fulfilled the criteria for giant prolactinoma and were included in the study. There was a male predominance with a male-to-female ratio of 8:1 (Table 1). The mean age at diagnosis was 47 (±16) years. The median PRL level was 6305 µg/L (range 1450-253 000). The hook effect was reported in 2 patients, in year 2001 and 2009, respectively, in both cases due to discordance between the PRL concentrations and the clinical and radiological findings. The median of the largest tumor diameter was 47 mm (range 40-85) (Table 1). The extension of the adenomas was assessed according to the SIPAP classification in 34 patients (25). In all but 1 patient, the tumors had invasive growth (parasellar grade ≥3 or infrasellar grade ≥1), and all tumors had suprasellar growth. None of the tumors were classified as carcinomas at diagnosis.

**Table 1.** Baseline characteristics of 84 patients with giant prolactinoma

	Entire cohort (n = 84)	Males (n = 75)	Females (n = 9)	*P-*value*
Age (years)	47 (±16)	47 (±16)	52 (±23)	.49
PRL (µg/L)	6305 (4095-11840)	6277 (4198-11792)	6836 (3969-12812)	.95
Tumor max diameter (mm)	47 (42-55)	47 (43-55)	41 (40-50)	.09
Visual field defects (%)	56/79 (71)	50/70 (71)	6 (67)	.77
Visual acuity impairment (%)	37/79 (47)	36/71 (51)	1/8 (13)	.06
Cranial nerve palsy (%)	7/67 (10)	5/58 (9)	2 (22)	.24
Headache (%)	36 (43)	31 (41)	5 (56)	.42
Galactorrhea (%)	4 (5)	3 (4)	1 (11)	.37
Apoplexy (%)	1 (1)	1 (1)	0	1.0
CSF leakage (%)	1 (1)	1 (1)	0	1.0
Hydrocephalus (%)	2 (2)	2 (3)	0	1.0
Incidentaloma (%)	8 (10)	6 (8)	2 (22)	.20
Pituitary insufficiency				
Gonadotrophin (%)	67/80 (84)	61/74 (82)	6/6 (100)	.26
TSH (%)	25/82 (30)	24/73 (33)	1/8 (13)	.26
ACTH (%)	14/81 (17)	12/72 (17)	2/8 (25)	.65
ADH	0	0	0	

Continuous data are presented as the mean ± standard deviation or median and interquartile range (Q1-Q3). Categorical data are presented as numbers and percentages, the latter calculated from those with available data.

Abbreviations: ADH, antidiuretic hormone; CSF, cerebrospinal fluid; PRL, prolactin.

*Comparison between sexes.

**Figure 1.**
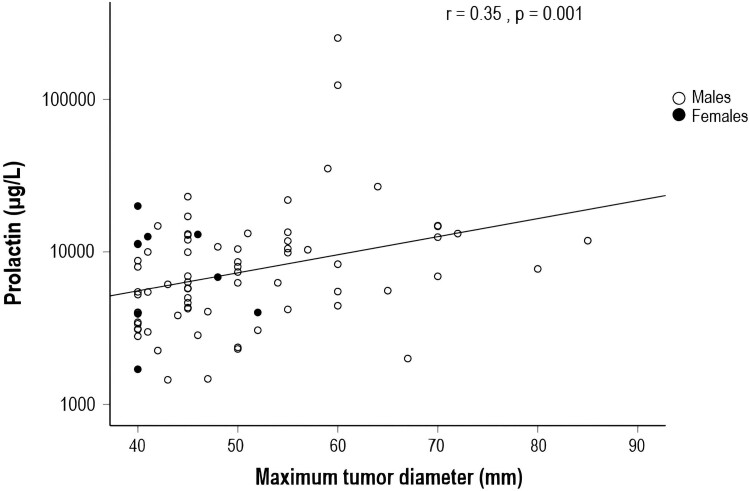
Correlation between PRL and maximum tumor diameter at baseline (n = 79), excluding 5 patients with serum PRL >5000 µg/L but with no information on the exact value.Abbreviations: PRL, prolactin.

The median duration of symptoms prior to diagnosis was 6 months (range 0-84), with no difference between sexes (*P* = .79). The most common clinical findings or symptoms at diagnosis were VFD (71%), reduced visual acuity (47%), and headache (43%). Spontaneous cerebrospinal fluid (CSF) rhinorrhea was reported in one patient at diagnosis. In 8 patients (10%) the diagnosis was made incidentally on CT or MRI of the brain. Gonadotropin deficiency was reported in 84% of the patients (67/80), TSH deficiency in 30% (25/82), and ACTH deficiency in 17% (14/81) (Table 1). None of the patients had antidiuretic hormone deficiency at diagnosis. There was a weak correlation between serum PRL and maximum tumor diameter at diagnosis (Spearman´s r = 0.35, *P* = .001) (Fig. 1).

### Treatment Modalities

**Figure 2.**
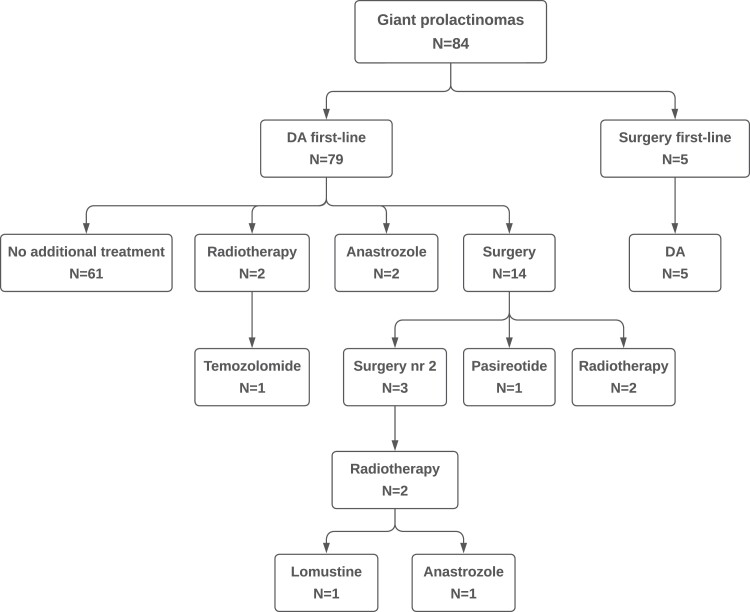
Flowchart of the treatment modalities in a cohort of 84 patients with giant prolactinomas.

All patients were treated with a DA at some point during the study period, either as primary treatment (n = 79) or after initial surgery (n = 5). The different therapeutic modalities are illustrated in Fig. 2. In the primary DA-treated patients, 23% (18/79) received additional therapy (surgery and/or radiotherapy and/or other medical therapy) after a median time of 14 months (IQR 4-29). In 14 patients, this was due to a lack of effect on PRL and/or tumor size, and in 4 patients, it was due to CSF leakage during DA treatment. Nineteen patients (23%) underwent pituitary surgery (13 with transsphenoidal, 6 with transcranial techniques). The proportion of patients who had surgery was similar over the time period [7/24 patients diagnosed from 1991-2004 vs 12/60 diagnosed from 2005-2018 (*P* = .36)]. Surgery was the primary treatment in 5 patients due to reduced visual acuity, VFD, or pituitary apoplexy. Three patients underwent reoperation. Six patients received radiotherapy [conventional radiotherapy (n = 4), gamma knife radiosurgery (n = 1), or proton radiation therapy (n = 1)]. Additional medical treatments were anastrozole (n = 3), pasireotide (n = 1), temozolomide (TMZ) (n = 1), and lomustine (n = 1).

The initial DA was bromocriptine (BRC) in 60% of the patients (n = 50), CAB in 38% (n = 32), and quinagolide in 2% (n = 2). During the first time period (1991-2004) the initial DA was BRC in 73% (16/22) of the patients and CAB in 27% (6/22), while from 2005 to 2018 BRC was initially given in 57% (34/60) and CAB in 43% (26/60) of the patients (*P* = .19). The median maximum dose of BRC was 5 mg/day (range 1.25-30), for CAB it was 2 mg/week (range 0.25-10.5), and for quinagolide it was 187 µg/day (range 75-450). Twenty-seven of 50 patients (54%) initially treated with BRC switched to CAB and 1 of 32 patients (3%) from CAB to BRC. At the last follow-up, 79 patients were treated with DA, 23% (n = 18) with BRC, 68% (n = 54) with CAB, and 9% (n = 7) with quinagolide.

Compared to the patients treated with DA monotherapy (n = 61), the patients who received multimodal treatment [DA + surgery and/or radiotherapy and/or other medical treatment (n = 23)] had larger tumors [maximum tumor diameter 55 mm (IQR 45-60) vs 45 mm (IQR 41-52) (*P* = .003)], had higher rates of VFD [91% vs 63% (*P* = .02)], and more frequently had TSH and ACTH deficiency at diagnosis [50% vs 23% and 33% vs 12% ,respectively (*P* = .02 for both comparisons)]. However, there were no significant differences in age at diagnosis, sex, or baseline serum PRL concentrations between the 2 groups.

### Overall Response to Treatment

The median follow-up after diagnosis was 9 years (IQR 4-15). At the last follow-up, the median serum PRL was 12 µg/L (IQR 4-126), with a median decrease in PRL of 99% (IQR 98-100) from diagnosis (*P* < .001). Forty-six patients (55%) had normalized PRL, and 54 (64%) had a PRL level <2×ULN (Table 2), with no difference between sexes (*P* = 1.0 and *P* = .48, respectively). At the last follow-up, the median maximum tumor diameter had decreased to 22 mm (IQR 3-40), with a median tumor diameter reduction of 50% (IQR 23-94) (*P* < .001). Tumor response was reported in 58 patients (69%), of whom 16 had complete tumor regression on MRI. Tumor enlargement was observed in 1 patient. A combined response occurred in 36 patients (43%) (Table 2). In 49 patients with VFD at diagnosis and available follow-up data, an improvement was observed in 82% of the patients (n = 40), no change in 14% (n = 7), and deterioration in 4% (n = 2) (Table 2). Among 33 patients with reduced visual acuity at diagnosis, the corresponding percentages were 73% (n = 24), 21% (n = 7), and 6% (n = 2), respectively.

**Table 2.** Treatment outcomes at the last follow-up in the whole cohort and in subgroups of patients with DA) monotherapy vs multimodal treatment

	Entire cohort (n = 84)	DA monotherapy (n = 61)	Multimodal treatment (n = 23)	*P*-value*
Follow-up time (years)	9 (4-15)	9 (4-15)	8 (5-17)	.90
Final PRL (µg/L)	12 (4-126)	8 (4-52)	128 (8-1002)	.01
PRL normalization (%)	46 (55)	38 (62)	8 (35)	.02
PRL <2×ULN (%)	54 (64)	44 (72)	10 (43)	.02
Tumor max diameter (mm)	22 (3-40)	16 (2-37)	31 (2-43)	.24
Tumor disappearance (%)	16 (19)	12 (20)	4 (17)	1.0
Tumor response (incl disappearance) (%)	58 (69)	41 (67)	17 (74)	.55
Tumor enlargement (%)	1 (1)	0	1 (4)	.27
Combined response (PRL normal and tumor response) (%)	36 (43)	29 (48)	7 (30)	.16
Visual fields defects				
Improved (%)	40/49 (82)	25/30 (83)	15/19 (79)	.70
Deteriorated (%)	2/49 (4)	0/30	2/19 (11)	.15
Pituitary insufficiency				
Gonadotrophin (%)	59/80 (74)	39/58 (67)	20/22 (91)	.03
TSH (%)	36/83 (43)	20/60 (33)	16 (70)	.003
ACTH (%)	19/82 (23)	8/59 (14)	11 (48)	.001
ADH (%)	3/83 (4)	0/60	3 (13)	.02

Continuous data are presented as the median and interquartile range (Q1-Q3). Categorical data are presented as numbers and percentages, the latter calculated from those with available data. Multimodal treatment includes treatment with DA plus surgery and/or radiotherapy and/or other medication.

Abbreviations: ADH, antidiuretic hormone; DA, dopamine agonist; PRL, prolactin; ULN, upper level of normal.

*Comparison between DA monotherapy and multimodal treatment.

At the last follow-up, 74% (59/80) of the patients had gonadotropin deficiency (Table 2). Among the patients with available data both at baseline and at the last follow-up (n = 65), gonadal function recovered in 11 (17%), and all of these patients were treated with DA monotherapy. However, hypogonadism developed in 4 initially eugonadal patients, 2 of whom were treated with surgery and 2 with DA monotherapy. Forty-six men with hypogonadism received testosterone replacement after diagnosis. After initiating testosterone replacement, the serum PRL increased in 3 patients. Two of them required a higher DA dose, while anastrozole was added in 1, and these treatments led to a decrease in PRL in all 3. None of the other 43 men who received testosterone replacement had an increase in PRL that required a change in treatment of the prolactinoma. Two women with hypogonadism (age at diagnosis of 23 and 35 years) received estrogen replacement after diagnosis, and in both patients their serum PRL remained unchanged.

### DA Monotherapy

**Figure 3.**
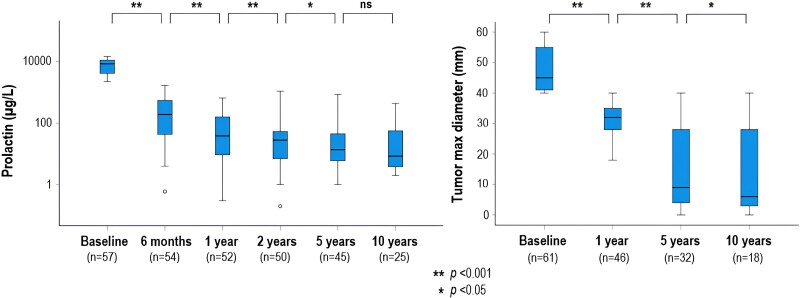
PRL and maximum tumor diameter over time in 61 patients with giant prolactinoma treated with dopamine agonist monotherapy, excluding 4 patients with serum PRL >5000 µg/L at baseline but with no information on the exact value.Abbreviations: PRL, prolactin.

In 61 patients treated with DA monotherapy, the median serum PRL level decreased from 6274 µg/L (IQR 4013-11 557) at diagnosis to 8 µg/L (IQR 4–52) at the last follow-up (*P* < .001) and the median maximum tumor diameter decreased from 45 mm (IQR 41-52) to 16 mm (IQR 2-37) (*P* < .001). The combined response was 48% (n = 29). The decrease in PRL and tumor size over time is shown in Fig. 3. Thirty-nine patients treated with DA monotherapy were on CAB at last follow-up; among them PRL normalized in 56% (n = 22). In this group, the median weekly dose of CAB was 1.5 mg (range 0.75-7.0) compared to 3.0 mg (range 0.25-7.0) in the patients with nonnormalized PRL (n = 17) (*P* = .15). Among 33 patients who received a CAB dose ≥2.0 mg/week, 36% (n = 12) were considered DA responders (normalized PRL with CAB <2 mg/week), 30% (n = 10) had partial DA resistance (normalized PRL with CAB ≥2.0 mg/week), and 33% (n = 11) had DA resistance (elevated PRL despite CAB ≥2.0 mg/week). In 2 patients achieving PRL normalization and complete tumor regression after a total treatment period of 3 and 21 years, respectively, DA (BRC and CAB, respectively) was discontinued. At the last follow-up, 6 and 36 months after DA withdrawal, PRL remained normal.

### DA Monotherapy vs Multimodal Treatment

The patients who received multimodal treatment (n = 23) had higher median PRL (*P* = .01) and lower rates of PRL normalization (*P* = .02) at the last follow-up than the patients with DA monotherapy (n = 61) (Table 2). Additionally, the patients who received multimodal treatment had higher rates of pituitary deficiencies [hypogonadism (*P* = .03), hypothyroidism (*P* = .003), hypocortisolism (*P* = .001), diabetes insipidus (*P* = .02)] at the last follow-up (Table 2).

### Immunohistochemistry and Ki-67 Index

Immunohistochemical analysis of the tumors was available in 16/19 patients who underwent surgery. PRL immunoreactivity was demonstrated in 15 tumors (1 was not analyzed due to technical problems). The median value of the proliferation marker Ki-67 analyzed in 14 tumors was 4.5% (range 1-40). The highest value at 40% was observed in the hot spots of a tumor, and the second highest value was 15% in 2 patients. Eight tumors had Ki-67 > 3%, and 4 had Ki-67 ≥ 10%. There was no difference in the median maximum tumor diameter at diagnosis in the tumors with Ki-67 > 3% compared to the tumors with Ki-67 ≤ 3% [54 mm (IQR 45-60) vs 58 mm (IQR 45-62), (*P* = .74)]. None of the 8 patients with tumor Ki-67 > 3% achieved a combined response at the last follow-up, whereas 4 of the 6 patients (67%) with tumor Ki67 ≤ 3% did (*P* = .02). Three patients were considered to fulfill the definition for aggressive pituitary tumors [invasiveness and unusually rapid growth despite standard treatment (DA, surgery, radiotherapy)] (26).

### Prediction of Treatment Response

In patients treated primarily with DA (n = 79), reduction of serum PRL to <2×ULN or reduction of the maximum tumor diameter by 20% after the first year of treatment were both associated with a combined response at the last follow-up [both univariate and multivariate (Table 3) analyses]. Age, sex, VFD at diagnosis, and multimodal treatment were not associated with a combined response (data not shown). PRL levels, maximum tumor diameters at diagnosis, and multimodal treatment did not predict combined response at the last follow-up (Table 3).

**Table 3.** Multivariate analysis of the variables associated with combined biochemical and tumor response at the last follow-up in patients with primary dopamine agonist treatment (n = 79)

	OR (95% CI)	*P*-value
Initial serum PRL	1.00 (1.00-1.00)	.66
Initial max tumor diameter	0.98 (0.89-1.1)	.62
Multimodal treatment	0.21 (0.02-1.9)	.16
PRL <2xULN at year 1	30.5 (4.9-189.8)	<.001
Reduction of max tumor diameter ≥20% at year 1	14.8 (1.8-122.9)	.012

Abbreviations: CI, confidence interval; OR, odds ratio; PRL, prolactin; ULN, upper level of normal.

## Discussion

Giant prolactinomas are rare, and treatment can be a challenge in some patients. In this nationwide study of 84 patients with giant prolactinomas followed for a median of 9 years after diagnosis, most of the patients had a good outcome of treatment, with long-term normalization of PRL achieved in over half of the patients and a significant tumor response in almost 70%. The largest decrease in PRL levels occurred during the first year of DA treatment, and the long-term combined response was predicted by the effect after the first year of treatment. Most of the patients were treated with DA monotherapy, but approximately 1 in 4 needed additional therapy, mainly pituitary surgery.

A large majority of the patients in the current study were men, and the most frequent symptoms at diagnosis were related to the mass effects of the tumor, such as visual problems and headache, and these results are in line with the findings in smaller cohorts of giant prolactinomas (4, 12-14, 18, 27). In our study, 1 out of 10 patients were discovered incidentally on a brain CT/MRI that was undertaken for other reasons. Similar rates of incidentally diagnosed giant prolactinomas were observed in other studies (13, 14), indicating slow growth of the tumors over many years. In patients with prolactinomas, there is a correlation between PRL levels and tumor size at diagnosis (7, 15), an association that seems less evident in giant prolactinomas (r = 0.24-0.63) (4, 13, 18, 28). This is consistent with our study where a weak correlation between PRL levels and baseline maximum tumor diameters was observed (r = 0.35). When a patient presents with very high PRL levels, the diagnosis of a prolactinoma is evident. However, a discrepancy between tumor size and PRL levels warrants serial dilutions of PRL to exclude the “hook effect” (15). This was reported in 2/84 patients in our study, which is similar to other studies that reported the hook effect in 1/71 and 2/34 patients with giant prolactinomas, respectively (14, 29).

The overall treatment goals for prolactinomas are normalized PRL levels; recovery of gonadal, pituitary, and visual functions; and reduction of tumor size (22). However, in some patients with prolactinomas, these goals are not possible to reach, and the primary focus should be on tumor control and relieving compressive symptoms. In our cohort, PRL normalized in 55% of the patients, and a tumor response was seen in 69% of the patients. Accordingly, the normalization rate of PRL in giant prolactinomas treated with multimodal therapy in other studies was 44% to 65% (14, 18, 19, 21, 30). DAs are recommended as primary treatment in patients with prolactinomas, even for giant tumors with compressive symptoms (4, 15, 22, 31, 32). This is due to the potent effects of DAs, which in many cases can induce tumor shrinkage and relieve neurological symptoms within a few days (4, 33). In addition, complete surgical resection is rare (15, 22). The first-line treatment in our cohort was DA in all but 5 patients where surgery was the primary intervention due to visual disturbances or pituitary apoplexy. However, a complication of DA treatment is CSF leakage due to rapid tumor shrinkage. In our cohort, 4 patients (5%) developed CSF leakage during treatment with DAs, which led to additional treatment with temporary or permanent withdrawal of DAs. Other studies of giant prolactinomas have reported CSF leakage during DA treatment in 0% to 10% of patients (12-14, 27).

In the current study, a reduction in PRL to <2×ULN or tumor size by 20% in the first year of follow-up predicted a combined response at the last follow-up. This adds to the findings of a previous study where PRL reduction at the first month of BRC treatment significantly correlated with the nadir PRL at long-term follow-up (18). However, the knowledge of predicting the long-term outcomes of DA when this treatment is initiated is still limited. In this context, our result of the response to DA treatment is of clinical value. Thus, if a reduction in PRL or tumor size has not been obtained 1 year after initiation of DA treatment, a further decrease in PRL and/or tumor size may be protracted and/or insufficient, and in these patients a more thorough follow-up or multimodal treatment is warranted.

In our patients treated with DA monotherapy, PRL normalized in 62% of the patients, and there was a significant tumor response in 67%. In a review of 13 studies including 97 patients with giant prolactinomas primarily treated with DA, 60% had normalized PRL levels (ranging from 17% to 100% in different studies) and 74% had a significant tumor response (4). The responsiveness to DA appears to be similar in men and women (29), and in concordance we did not observe any differences between sexes. However, there were few women. In our study, 20% of the patients treated with DA monotherapy had no visible tumor on MRI at the last follow-up. This is in line with one study that reported tumor disappearance in 25% of 38 patients treated with DA monotherapy (14). The duration of DA treatment in patients with giant prolactinomas is expected to be life-long due to the potential risk of rapid tumor regrowth if therapy is discontinued (15, 32). In the literature, reports of successful withdrawal of DA in patients treated with DA monotherapy are scarce. In a case series, a successful withdrawal of DA was reported in 1 patient with a giant prolactinoma treated with DA monotherapy; however, the patient had an elevated ACTH level and possibly a mixed tumor (34). In 1 study, 2 out of 71 patients with giant prolactinomas maintained normal PRL and tumor response after DA withdrawal, but it is unclear whether these patients had received DA monotherapy or multimodal therapy (14). Two patients in the current study could discontinue DA treatment with maintained normoprolactinaemia; however, only 1 of them had a sufficient follow-up. Withdrawal of DA treatment in patients with giant prolactinomas should be an exception, and discontinuation of DA necessitates close surveillance of PRL and tumor size.

The most accepted definition of DA resistance is failure to normalize PRL levels with CAB doses from ≥1.5 to 2.0 mg per week and, in the case of macroprolactinoma, failure to achieve tumor size reduction of 30% to 50% (35-37). Resistance to CAB is more frequent in macro- compared to microprolactinomas (15-20% vs <10%) (36). In our cohort, hormonal resistance to CAB was observed in approximately one-third of the patients treated with DA monotherapy. In a review of patients with giant prolactinomas with primary DA treatment, 24% (18/76) were resistant to DA (daily dose of ≥15 mg BRC or a weekly dose of ≥2 mg CAB) (4); still, the overall normalization rate of PRL was similar to our study. However, it is important to consider that the DA dose escalation was at the treating physicians’ discretion, it cannot be excluded that use of higher DA doses might have reduced the number of patients resistant to DA. On the contrary, patients treated with DA monotherapy are probably a selection of patients that respond to DA. In the current study, it was not possible to evaluate resistance to BRC due to a switch to CAB in more than half of the patients.

The main hormonal consequence of hyperprolactinaemia is hypogonadotropic hypogonadism. In our study, as expected, most of the patients had hypogonadotropic hypogonadism at diagnosis, but only 17% had recovered their gonadal axis at the last follow-up. This is similar to data reported in other studies of giant prolactinomas (6, 13) but differs from a small study in which a recovery of gonadal function was reported in 67% (8/12) (38). Persistent hypogonadism despite a normalization of PRL obtained with DA may be the result of suppression of gonadotropins by longstanding hyperprolactinemia in combination with the mass effect of the tumor on normal pituitary tissue (22, 31, 32). In the current study, 85% of the males had testosterone replacement, which continued throughout the follow-up. However, since withdrawal of testosterone replacement in general was not attempted, we cannot exclude that resumption of gonadal function had occurred in some men. Testosterone aromatizes to estradiol, which might lead to lactotroph hyperplasia with an entailing increase in PRL and tumor size (39, 40). In our cohort, an increase in PRL was seen in 3 men after initiation of testosterone replacement and was addressed by an increased DA dose and/or by addition of the antiestrogen anastrozole. The initiation of replacement with sex hormones should be done with caution while monitoring PRL.

Pituitary surgery in giant prolactinomas is considered for the subset of patients with acute compressive symptoms due to pituitary apoplexy, progressive tumor growth, or visual deterioration not controlled with DA, as well as intolerance to DA treatment (5, 15, 22, 32). In the present cohort, surgery was performed in 6% of the patients as primary treatment and in 17% after the initial DA treatment. In the literature, the rates of surgery in giant prolactinomas vary considerably, between 13% and 62% (13, 14, 18-21). In our cohort, none of the patients who underwent surgery had normalized PRL postoperatively, and all needed additional treatment. In a study of 42 patients with giant prolactinomas, 48% (13/27) of the patients treated with BRC as primary treatment normalized PRL levels, in contrast to none who were primarily treated with surgery (n = 14) (18). At surgery, tissue for immunohistochemical analyses becomes available. In 2 studies of giant prolactinomas, the median Ki-67 was 2% and 5%, respectively (20, 28). In a cohort of giant prolactinomas with a maximum diameter ≥60 mm, the Ki-67 in 3 patients was 0.5% to 5% (30). From other observational studies of giant prolactinomas, information on Ki-67 is not available (12-14, 18, 21, 34, 37, 41). In our cohort, the median Ki-67 was high (4.5%), and more than half of the operated tumors had a Ki-67 > 3%. Our patients who underwent elective surgery were highly selected at multidisciplinary conferences, which might be an explanation for the high proliferation index. Patients with high Ki-67 need a thorough follow-up of tumor size. This is underlined by the fact that, in our study, none of the patients with Ki-67 > 3% achieved a combined response in contrast to 4 out of 6 of the patients with tumor Ki-67 ≤ 3%.

Six patients in our cohort received radiotherapy: 4 patients after surgery and 2 after DA. Three of these patients needed additional medical therapy after radiation. The risk of developing pituitary insufficiencies after radiotherapy and the long duration until a full effect is achieved are the disadvantages of radiotherapy (15, 36, 42). However, radiotherapy is an option due to its effect in controlling tumor growth (15). In our cohort, other adjuvant medical treatments were used in 6 patients (anastrozole, TMZ, lomustine, and pasireotide). TMZ is an oral alkylating agent, that has been recommended in the last decade as the first-line chemotherapy in the treatment of locally aggressive or malignant prolactinomas (26). In a study of 171 aggressive pituitary tumors and pituitary carcinomas, including 54 prolactinomas, tumor shrinkage was reported in 40% of 156 patients treated with TMZ (11). Another study reported an association between negative staining for the DNA repair protein O^6^-methylguanine DNA methyltransferase and a good response to TMZ (43). It is a challenge to find the right treatment for patients with giant prolactinomas not responding to DA, and additional treatment should always be discussed in a multidisciplinary team.

The limitations of the study are the retrospective design and the inborn issues with such a design, such as not having a predefined study protocol and missing data. On the other hand, our study reflects a real-life setting for this rare patient group. Other limitations are the use of different biochemical assays and lack of uniform re-evaluations of pituitary function. The strengths of our study are the large sample size, unselected nationwide cohort, long follow-up time, and detailed evaluation of each patient by experienced endocrinologists.

## Conclusion

In this nationwide study of a large cohort of patients with giant prolactinomas followed for a median of 9 years, treatment with DAs was effective and sufficient as monotherapy in most patients, and DAs could be withdrawn in 2 patients. A quarter of the patients needed additional treatment, mainly surgery, and 2 of 84 had an aggressive tumor course requiring chemotherapy. The decrease in PRL and tumor size after the first year of treatment predicted the long-term combined response. The study emphasizes the heterogeneity of these tumors and suggests that treatment response after 1 year can be useful for identifying patients who need a more careful follow-up and, in some cases, additional treatment.

## Data Availability

The datasets generated and/or analyzed during the current study are available from the corresponding author on reasonable request.
